# Supplementary figures and images for: Structure of the human ATM kinase and mechanism of Nbs1 binding
Source: eLife. 2022 Jan 25;11:e74218. doi: 10.7554/eLife.74218 (PMC8828054; doi:10.7554/eLife.74218)

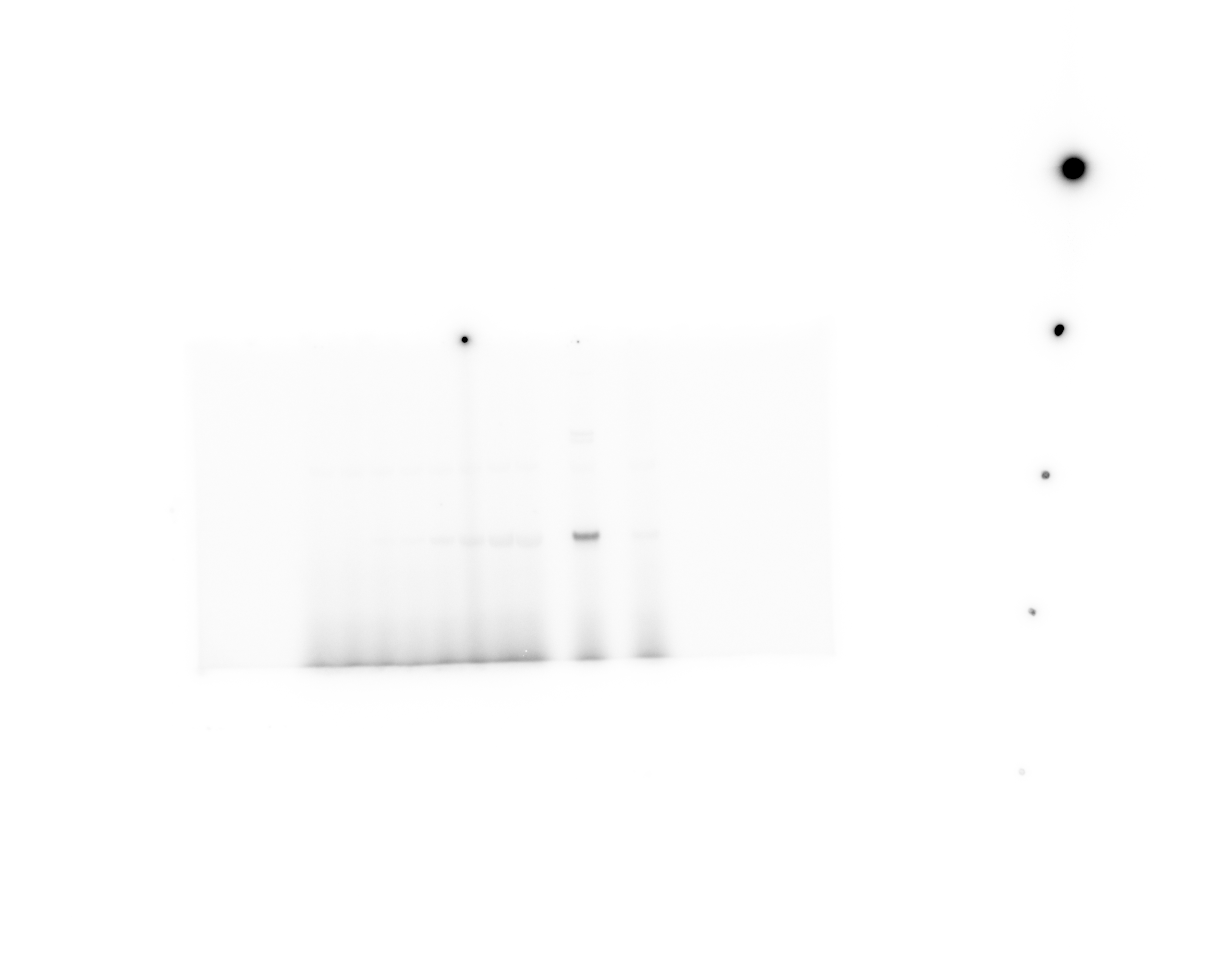

Supplement: Figure 1—figure supplement 1—source data 1. — Figure 1—figure supplement 1B ATM kinase assay without MRN or DNA. [file elife-74218-fig1-figsupp1-data1.zip › Figure 1 - figure supplement 1 - Source data/Figure 1 - figure supplement 1B.tif]

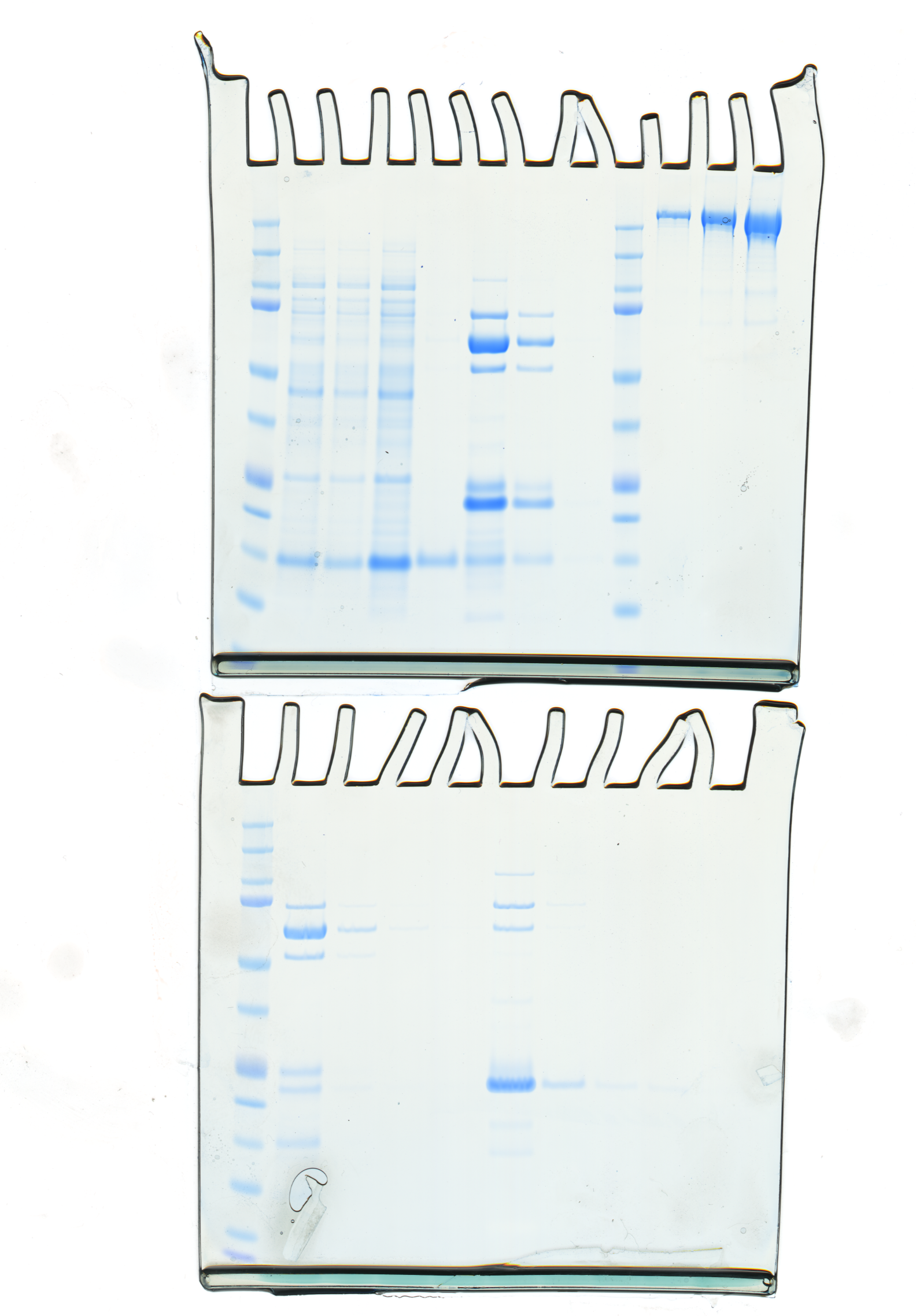

Supplement: Figure 1—figure supplement 1—source data 1. — Figure 1—figure supplement 1B ATM kinase assay without MRN or DNA. [file elife-74218-fig1-figsupp1-data1.zip › Figure 1 - figure supplement 1 - Source data/Figure 1 - figure supplement 1A.tiff]

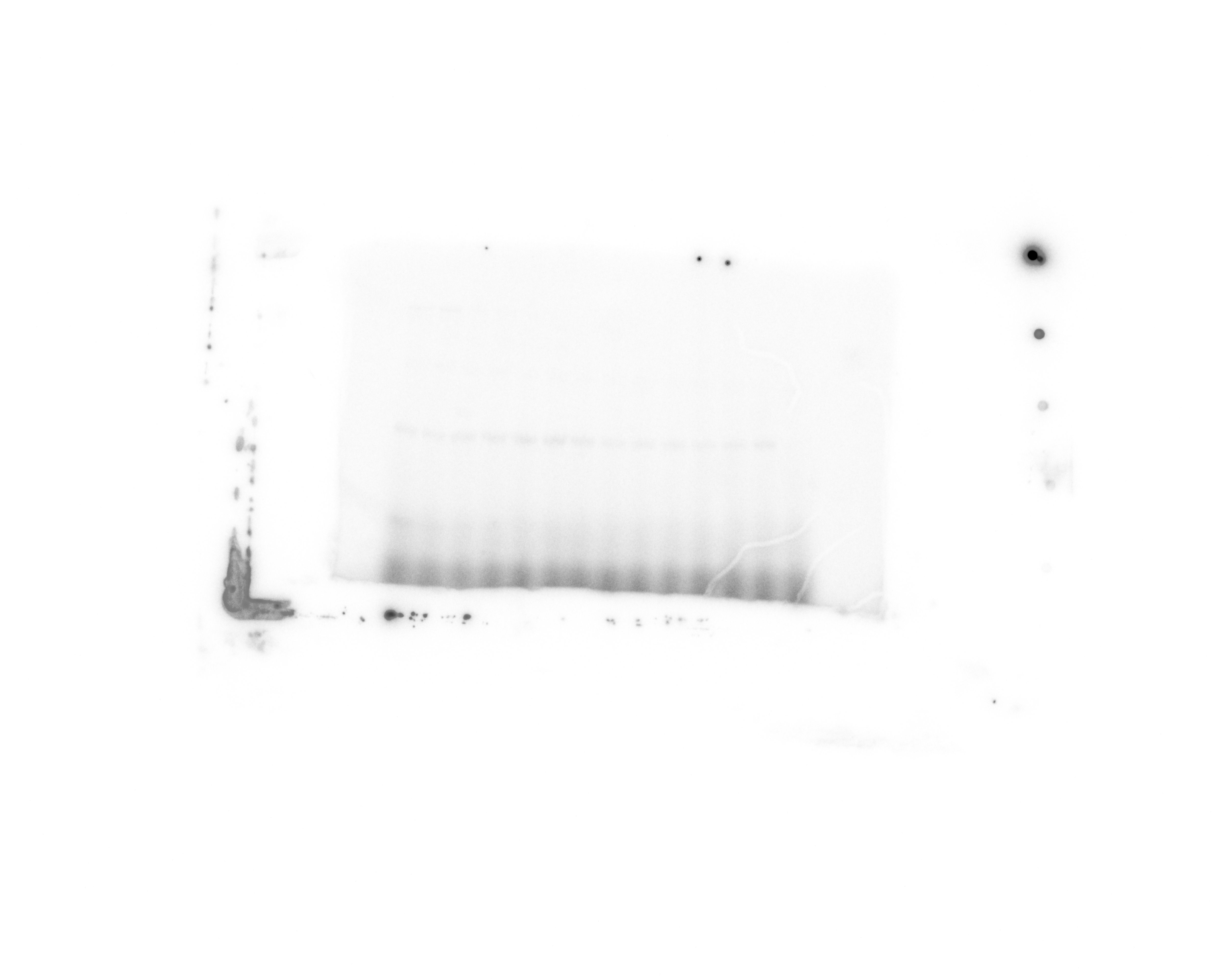

Supplement: Figure 4—figure supplement 2—source data 1. [file elife-74218-fig4-figsupp2-data1.zip › Figure 4 - figure supplement 2 - Source data/Figure 4 - figure supplement 2C.tif]

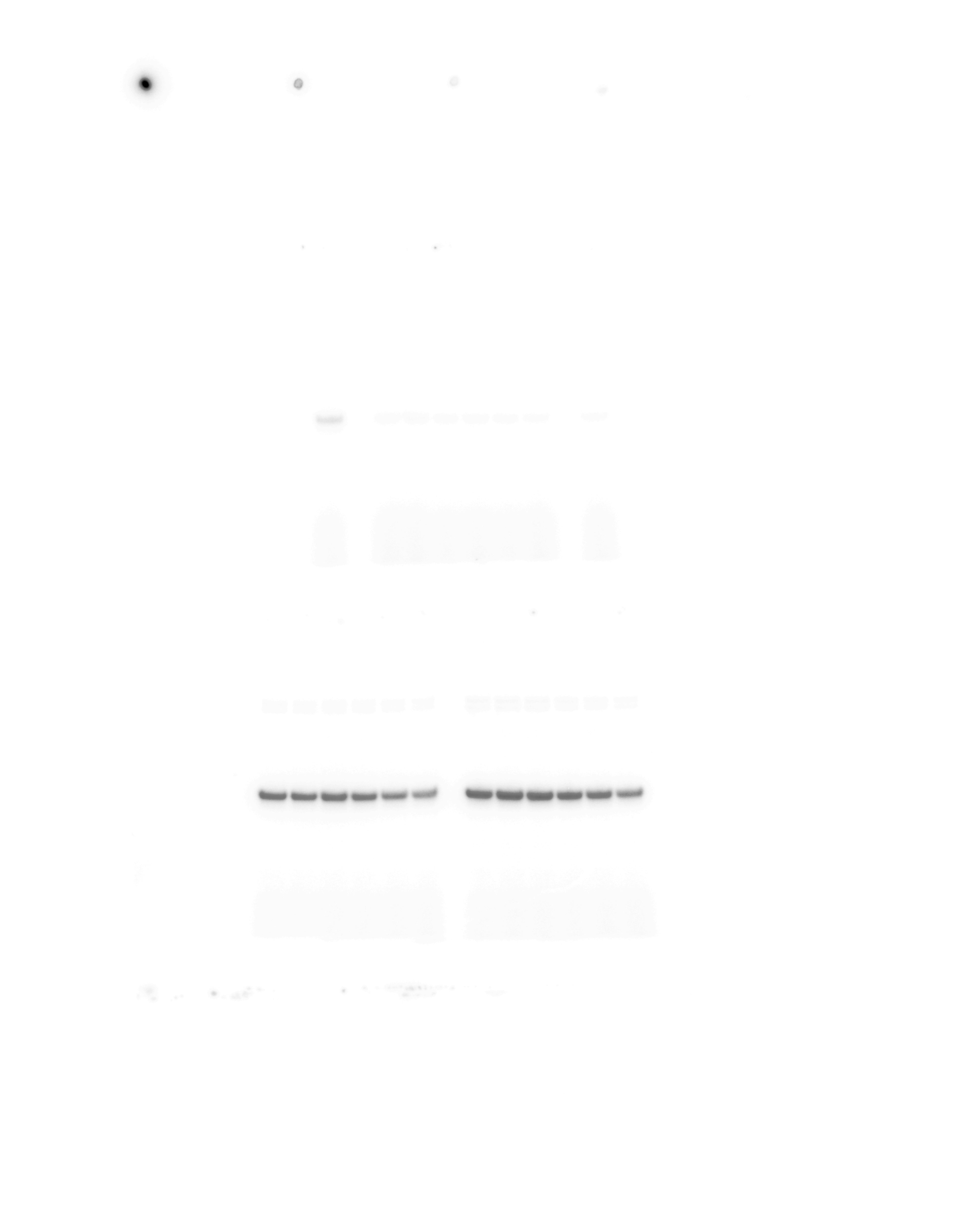

Supplement: Figure 5—source data 1. — Figure 5B ATM kinase assay with MRN and varying DNA concentration. Figure 5C ATM kinase assay with DNA and using wild-type MRN or FRY to AAA mutant. [file elife-74218-fig5-data1.zip › Figure 5 - Source data/Figure 5B.tif]

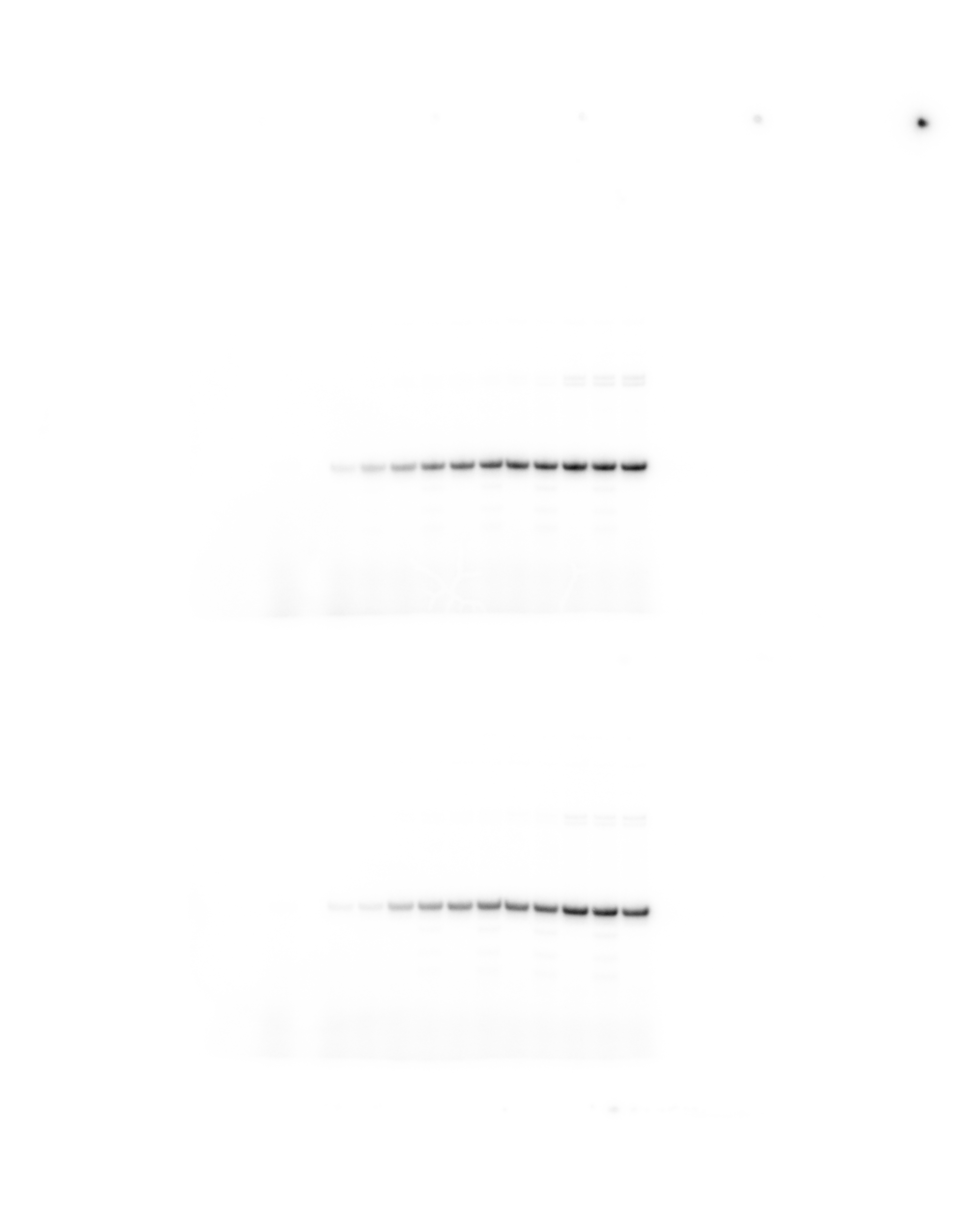

Supplement: Figure 5—source data 1. — Figure 5B ATM kinase assay with MRN and varying DNA concentration. Figure 5C ATM kinase assay with DNA and using wild-type MRN or FRY to AAA mutant. [file elife-74218-fig5-data1.zip › Figure 5 - Source data/Figure 5C.tif]

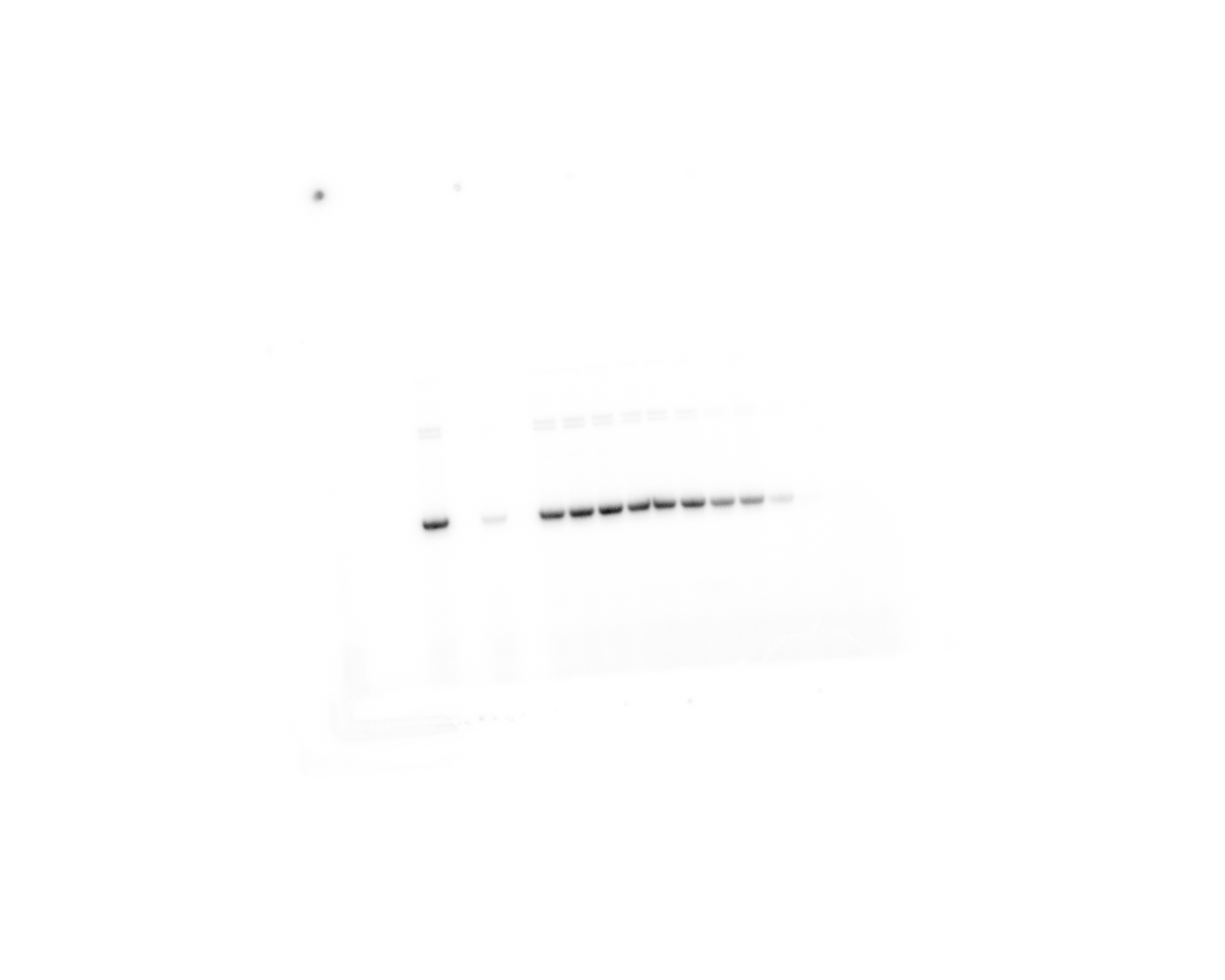

Supplement: Figure 5—source data 1. — Figure 5B ATM kinase assay with MRN and varying DNA concentration. Figure 5C ATM kinase assay with DNA and using wild-type MRN or FRY to AAA mutant. [file elife-74218-fig5-data1.zip › Figure 5 - Source data/Figure 5A.tif]

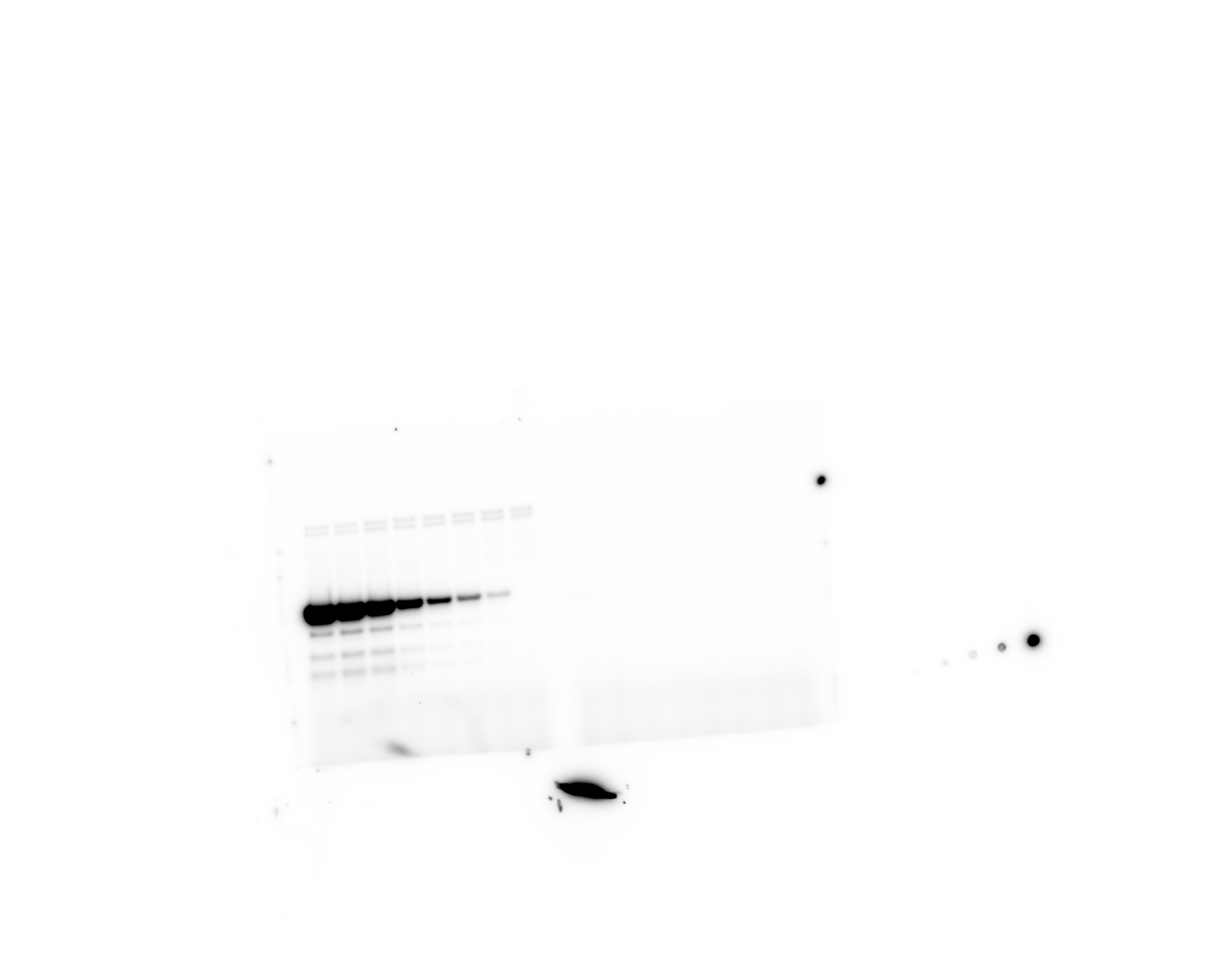

Supplement: Figure 5—figure supplement 1—source data 1. — Figure 5—figure supplement 1B ATM kinase assay with MRN and without DNA. Figure 5—figure supplement 1C ATM kinase assay with DNA and MRN, without and with ATM. Figure 5—figure supplement 1E ATM kinase assay with DNA, without and with MRN. Figure 5—figure supplement 1G Single-turnover ATM kinase assay. [file elife-74218-fig5-figsupp1-data1.zip › Figure 5 - figure supplement 1 - Source data/Figure 5 - figure supplement 1E.tif]

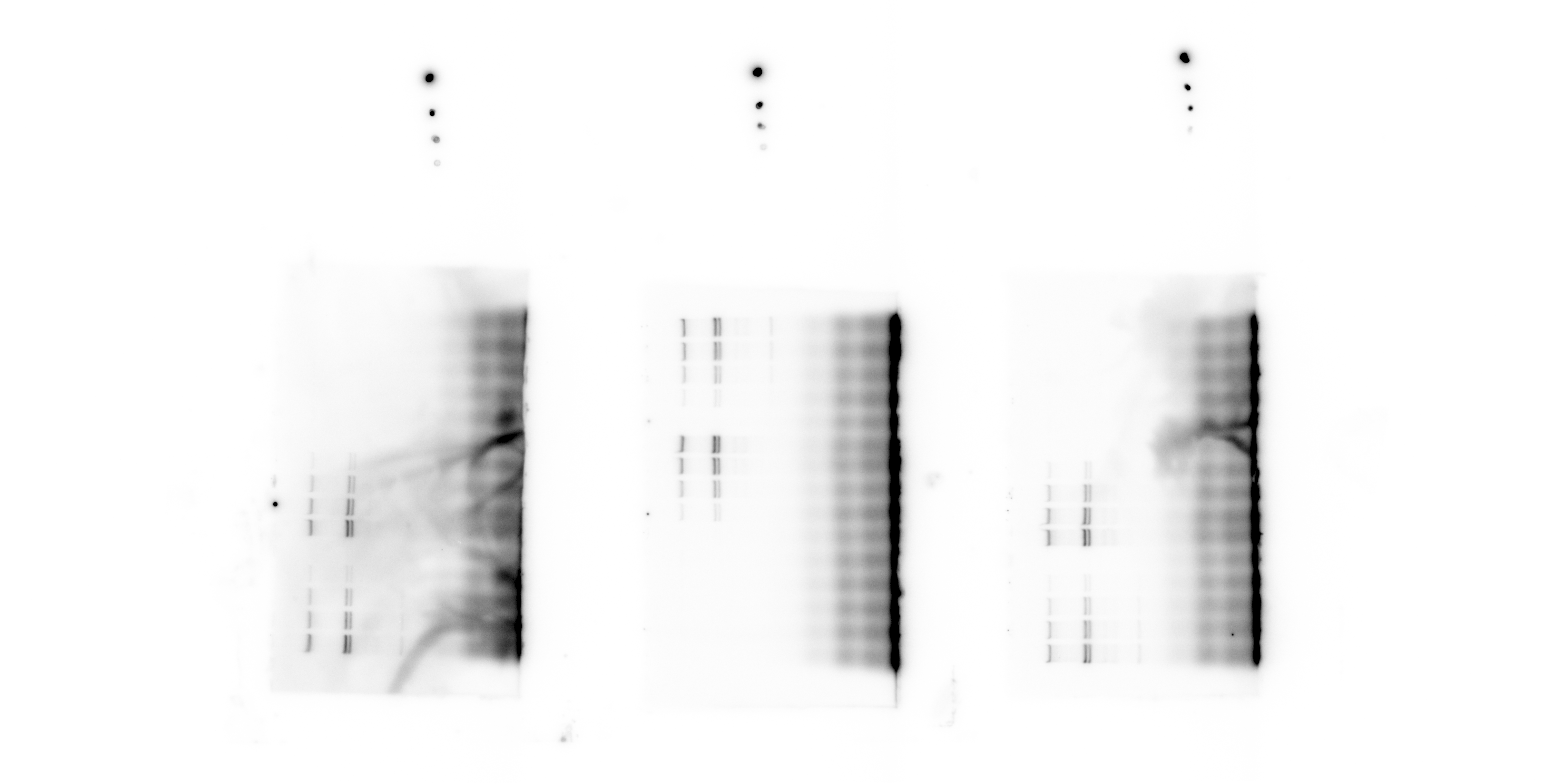

Supplement: Figure 5—figure supplement 1—source data 1. — Figure 5—figure supplement 1B ATM kinase assay with MRN and without DNA. Figure 5—figure supplement 1C ATM kinase assay with DNA and MRN, without and with ATM. Figure 5—figure supplement 1E ATM kinase assay with DNA, without and with MRN. Figure 5—figure supplement 1G Single-turnover ATM kinase assay. [file elife-74218-fig5-figsupp1-data1.zip › Figure 5 - figure supplement 1 - Source data/Figure 5 - figure supplement 1G.tif]

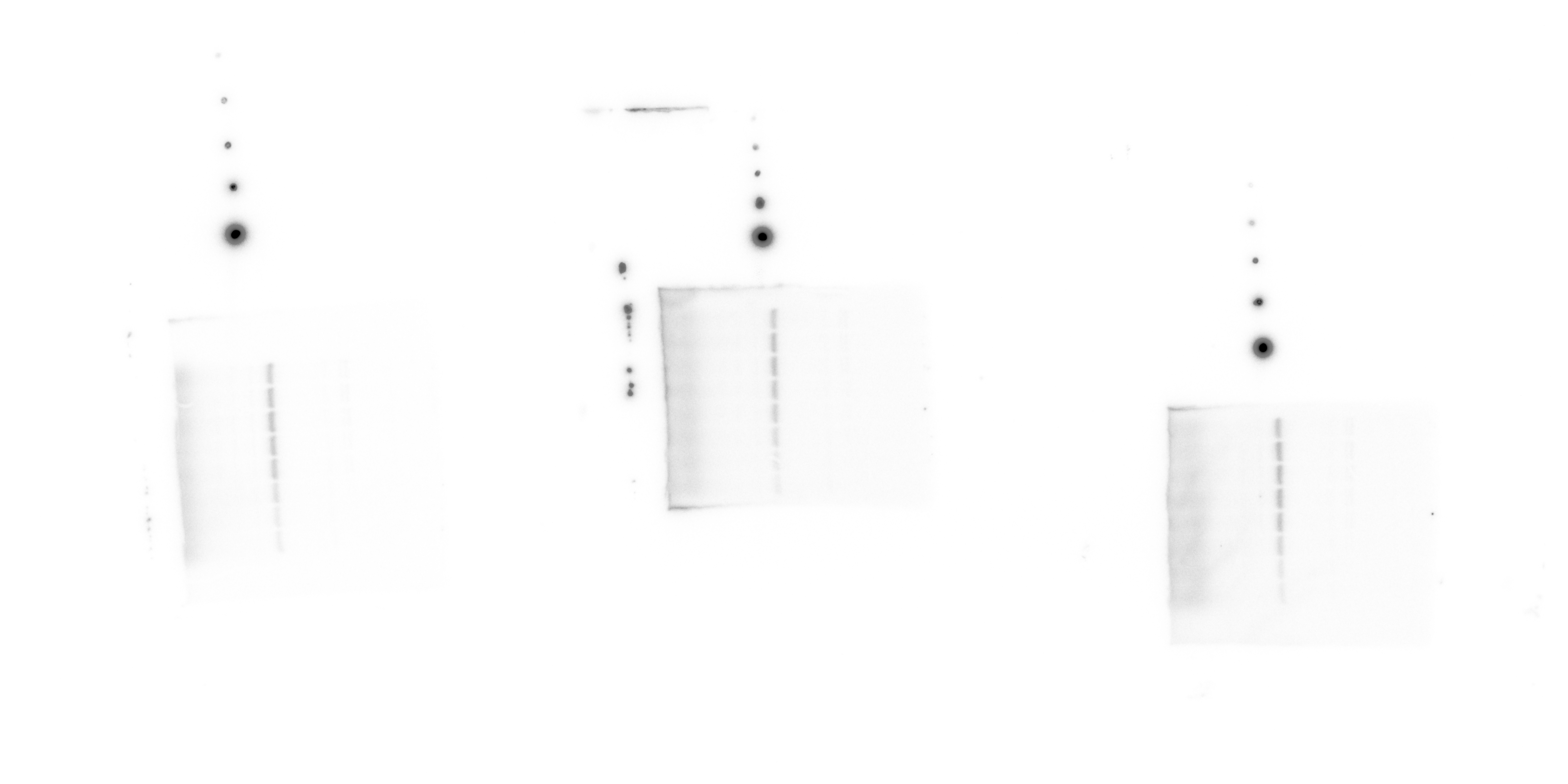

Supplement: Figure 5—figure supplement 1—source data 1. — Figure 5—figure supplement 1B ATM kinase assay with MRN and without DNA. Figure 5—figure supplement 1C ATM kinase assay with DNA and MRN, without and with ATM. Figure 5—figure supplement 1E ATM kinase assay with DNA, without and with MRN. Figure 5—figure supplement 1G Single-turnover ATM kinase assay. [file elife-74218-fig5-figsupp1-data1.zip › Figure 5 - figure supplement 1 - Source data/Figure 5 - figure supplement 1B.tif]

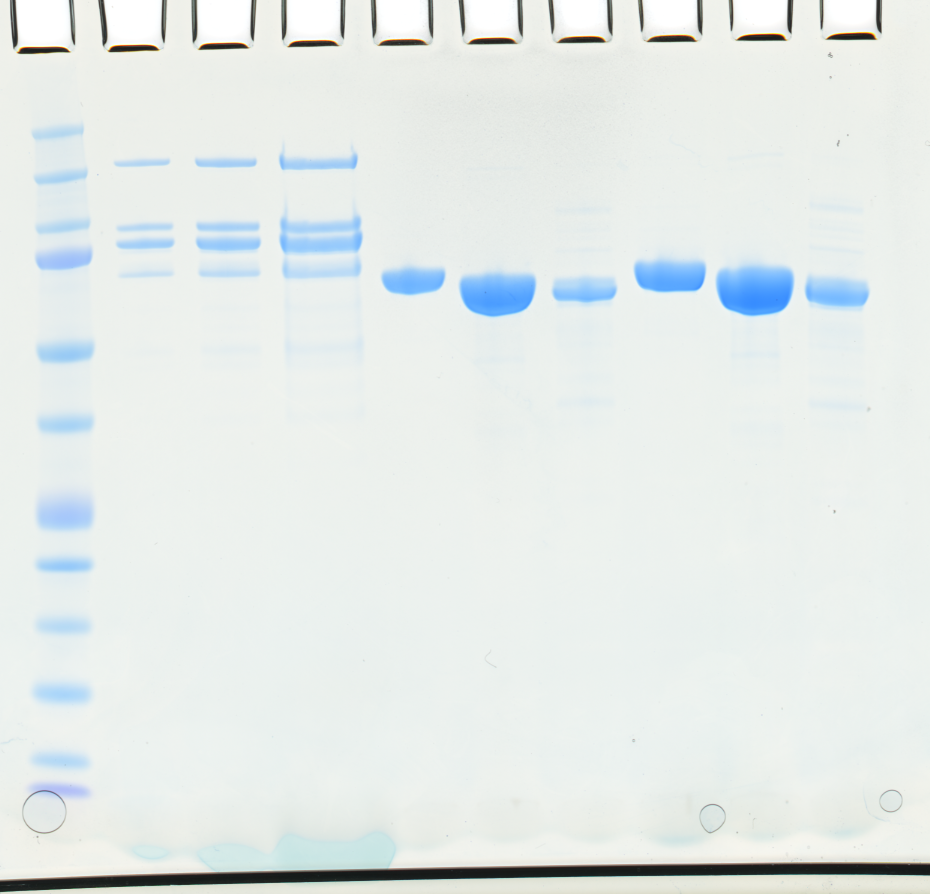

Supplement: Figure 5—figure supplement 1—source data 1. — Figure 5—figure supplement 1B ATM kinase assay with MRN and without DNA. Figure 5—figure supplement 1C ATM kinase assay with DNA and MRN, without and with ATM. Figure 5—figure supplement 1E ATM kinase assay with DNA, without and with MRN. Figure 5—figure supplement 1G Single-turnover ATM kinase assay. [file elife-74218-fig5-figsupp1-data1.zip › Figure 5 - figure supplement 1 - Source data/Figure 5 - figure supplement 1A.tiff]

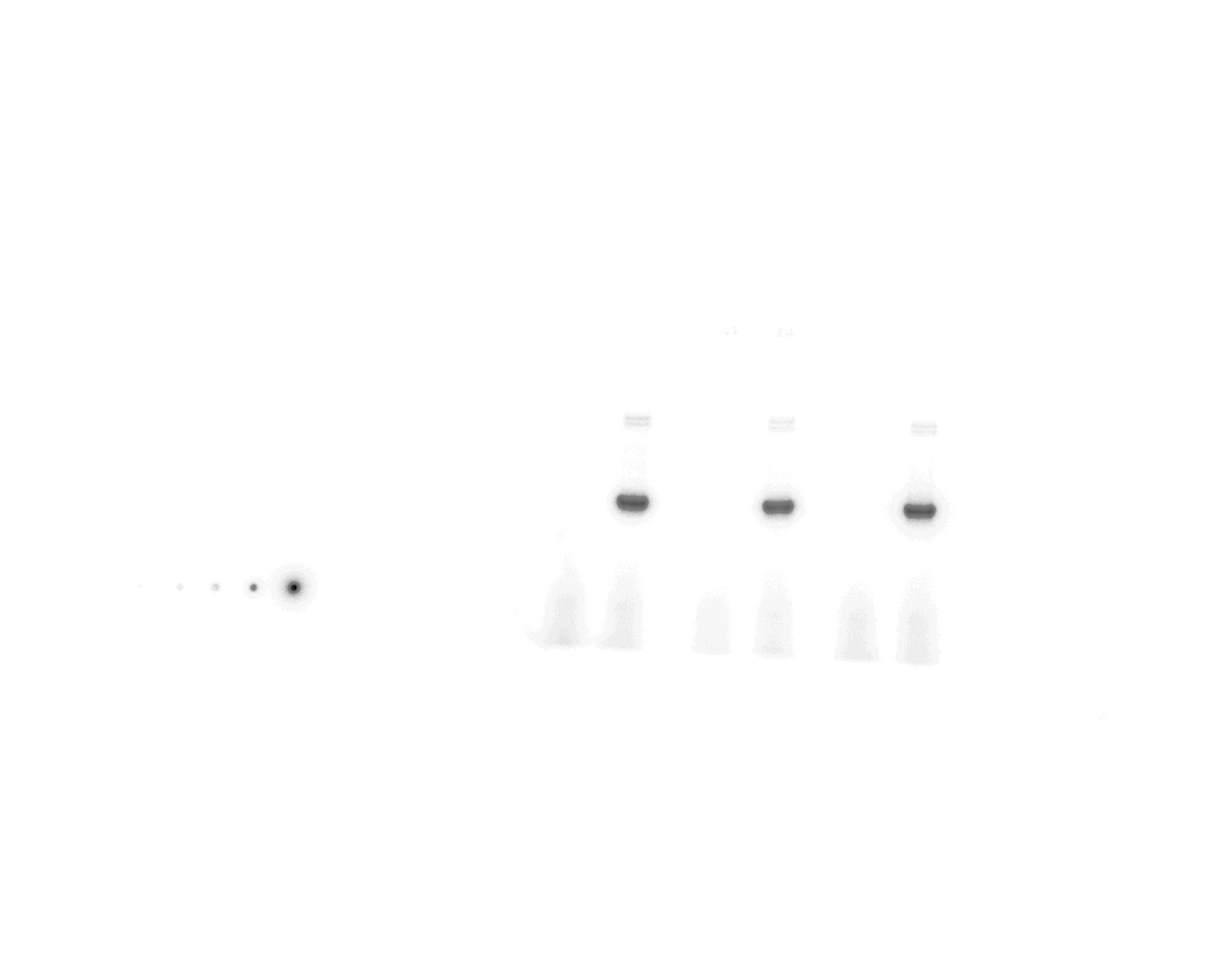

Supplement: Figure 5—figure supplement 1—source data 1. — Figure 5—figure supplement 1B ATM kinase assay with MRN and without DNA. Figure 5—figure supplement 1C ATM kinase assay with DNA and MRN, without and with ATM. Figure 5—figure supplement 1E ATM kinase assay with DNA, without and with MRN. Figure 5—figure supplement 1G Single-turnover ATM kinase assay. [file elife-74218-fig5-figsupp1-data1.zip › Figure 5 - figure supplement 1 - Source data/Figure 5 - figure supplement 1C.tif]
