# Supplementary material for: Structure of the human ATM kinase and mechanism of Nbs1 binding
Source: eLife. 2022 Jan 25;11:e74218. doi: 10.7554/eLife.74218 (PMC8828054; doi:10.7554/eLife.74218)

Figure 4E

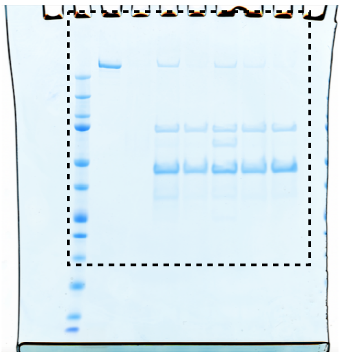

Figure 5A

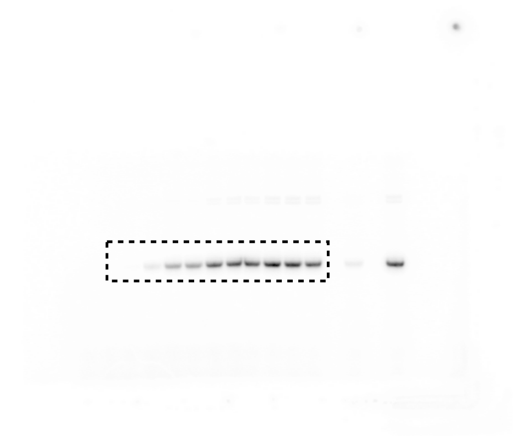

Figure 5B

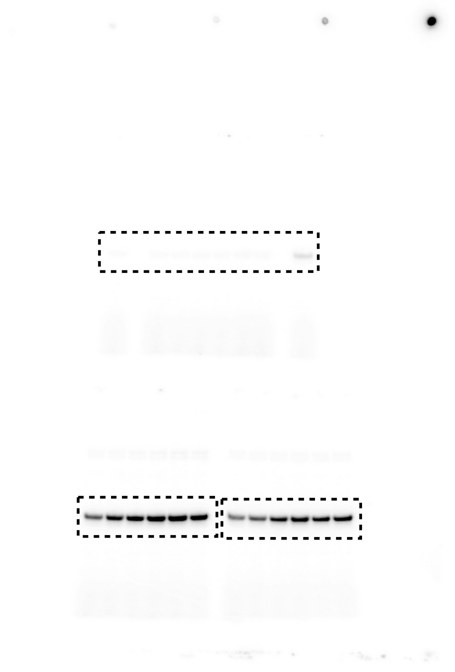

Figure 5C

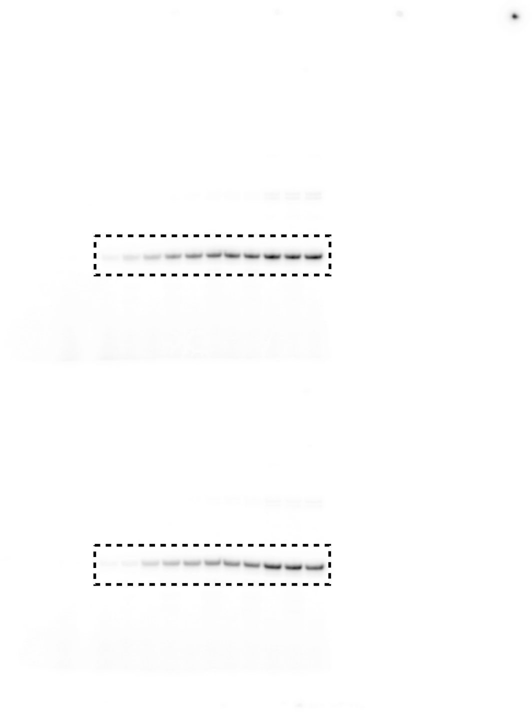

Figure 1 - Figure Supplement 1A

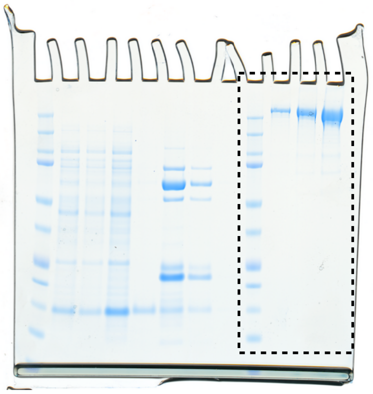

Figure 1 - Figure Supplement 1B

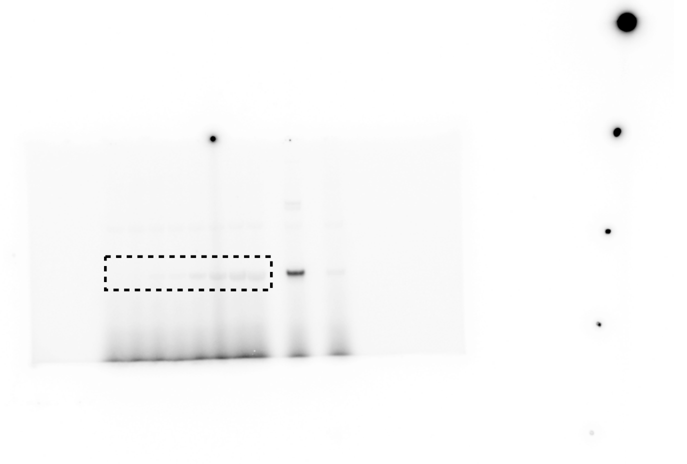

Figure 4 - Figure Supplement 2C

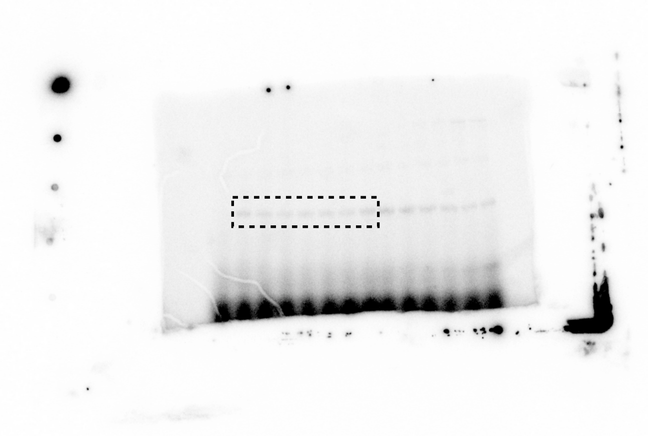

Figure 5 - Figure Supplement 1A

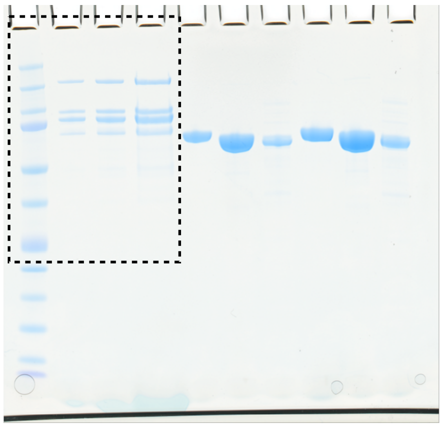

Figure 5 - Figure Supplement 1E

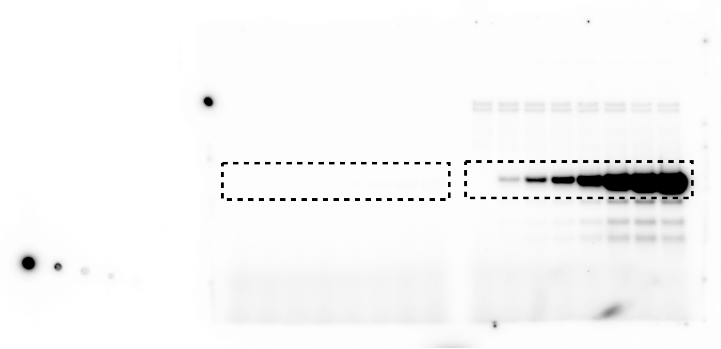

Figure 5 - Figure Supplement 1B

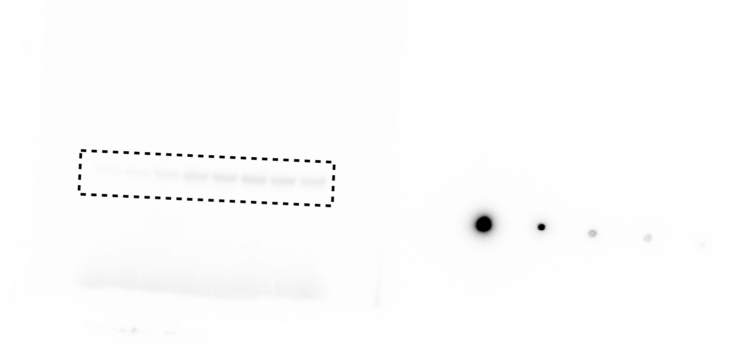

Figure 5 - Figure Supplement 1G

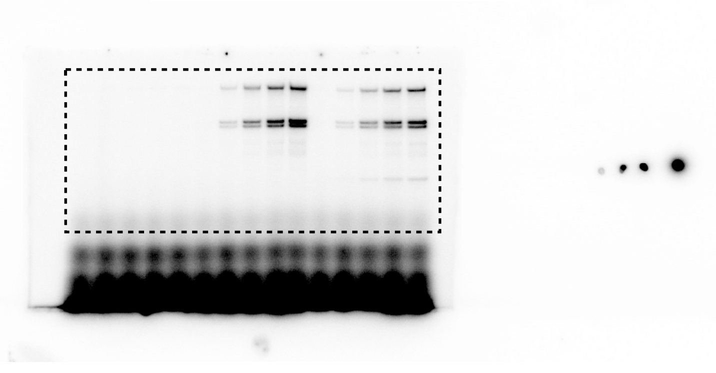

Figure 5 -
Figure Supplement 1C

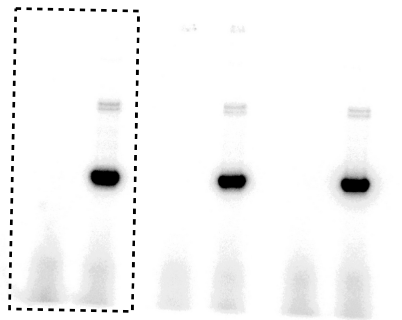

Supplement: Source data 1. [file elife-74218-data1.pdf]
